# How much obesity and diabetes do impair male fertility?

**DOI:** 10.1186/s12958-022-01034-w

**Published:** 2023-05-19

**Authors:** Shima AbbasiHormozi, Azam Kouhkan, Abdolhossein Shahverdi, Amir Parikar, Azin Shirin, Samira Vesali

**Affiliations:** 1 Reproductive Epidemiology Research Center, Royan Institute for Reproductive Biomedicine, ACECR, Tehran, P.O. Box: 16635-148 Iran; 2 Department of Embryology, Reproductive Biomedicine Research Center, Royan Institute for Reproductive Biomedicine, ACECR, Tehran, Iran; 3 Department of Andrology, Reproductive Biomedicine Research Center, Royan Institute for Reproductive Biomedicine, ACECR, Tehran, Iran; 4 Faculty of Sport Science & Health, Shahid Beheshti University, Tehran, Iran

**Keywords:** Sub fertility, Obese men, Diabetic men, Metabolic dysfunction, Hormonal dysfunction, Inflammatory

## Abstract

**Background:**

Subfertility in obese and diabetic men during the reproductive age is evident, but the mechanisms by which obesity and diabetes mellitus cause male infertility are not entirely understood**.** The current study aimed to evaluate the effects and potential mechanisms of obesity and diabetes on male fertility.

**Methods:**

We enrolled control = 40, obese = 40, Lean-DM = 35, and Obese-DM = 35 individuals. The obesity-associated markers, diabetic markers, hormonal and lipid profile, inflammatory indices, and semen analysis were assessed in four experimental groups.

**Results:**

Our finding showed that diabetic markers were significantly increased in two diabetic groups, while obesity indices were markedly increased in two obese groups. Conventional sperm parameters were significantly lower in three groups compared with the control. Serum levels of total testosterone and sex hormone-binding globulin were significantly lower in men with obesity and DM compared with the control. There was a significant difference in the concentration of high-sensitivity C-reactive protein among four experimental groups. Moreover, serum leptin was significantly increased in obese DM, lean DM, and obese groups. Serum insulin levels had a positive correlation with metabolic-associated indices and high-sensitivity C-reactive protein levels, whereas it had a negative correlation with count, motility, and morphology.

**Conclusions:**

Our findings showed the metabolic changes, hormonal dysfunction and inflammatory disturbance might be suspected mechanisms of subfertility in obese and diabetic subfertile men.

## Background

Obesity and type 2 diabetes mellitus (DM) are two known metabolic disorders with an increasing prevalence and high mortality rate [1]. Metabolic disorders are characterized by hyperglycemia, hyperlipidemia, and insulin resistance, increasing the risk of cardiovascular diseases and premature death [2]. Approximately half of the patients with type 2 diabetes are obese. The increasing incidence of diabetes can thus be attributed to the global epidemic of obesity.

Alongside an increased incidence in diabetes and obesity, infertility is a growing concern affecting up to 15% of couples trying to conceive globally. Recently, the effects of obesity and diabetes on fertility have been extensively investigated [2–4]. Although reproductive dysfunction and declined quality of semen were reported in obesity and diabetes [5], but There is are no clear studies of the involved molecular signaling pathways in obese and diabetic-related infertility complications and the mechanisms by which obesity and diabetes mellitus (DM) causes male infertility are not clearly explained [6–8].

Multiple pathways have been proposed for the decline in male fertility in obese and diabetes men. These histopathological changes could be the result of impaired Hypothalamus Pituitary Gonadal (HPG) Axis [9–11], increased reactive oxygen species (ROS) and DNA fragmentation of spermatozoa [12, 13], biochemical disturbance [14, 15], Several lines of evidence have shown the role of hormonal and antioxidant in sub fertility following diabetes and obesity [16]. Besides, the role of inflammatory markers in the development of diabetes mellitus and obesity have been addressed [17] [18]. This study aimed to determine the impact of obesity and diabetes, alone or combined that may affect male reproductive functions.

## Materials and methods

### Data collection

In this case–control study, 150 subjects with the age range of 30–50 years old were selected from the Royan Institute Infertility Center (a referral infertility clinic in Tehran, Iran) They were divided into the following groups:

1. Control group: consisted of men with normal weight who were non-diabetic
2. Lean DM group: included men with normal weight who were afflicted with diabetes
3. Obese group: comprised of obese men who were non-diabetic
4. Obese-DM group: consisted of obese men who were affected by diabetes mellitus

The experimental procedure of the current research was approved by the Ethics Committee of Royan Institute (No. IR.ACECR.ROYAN.REC.1396.53). Informed consent was obtained from all individuals who voluntarily participated in our study. The questionnaire was completed for each participant, including demographic characteristics, medical and drug histories, smoking status, alcohol consumption, sexual activity, and surgical history. Males with a history of azoospermia, urogenital infections, varicocele, chronic severe debilitating medical illness (cerebrovascular, cardiovascular, sexually transmitted diseases, systemic disorders, and acute infections) were excluded from the study.

## Anthropometrical measure

After enrollment, all participants underwent physical examinations that included anthropometric measurements [body height and weight, waist circumference (WC), and hip circumference (HC)]. The body mass index (BMI) was classified into normal weight (BMI 18–25 kg/m2) and obese (BMI ≥ 30 kg/m2). Waist circumference, hip circumference, and the waist to hip ratio (WHR) were also measured. Diabetes mellitus was diagnosed according to the diagnostic criteria provided by the American Diabetes Association [19].

Body height and weight were determined for each participant standing without shoes and heavy outer garments. Waist circumference was calculated at the level midway between the lower rib margin and the iliac crest of participants in a standing position without heavy outer garments and with emptied pockets, breathing out gently. Hip circumference was recorded as the maximum circumference over the buttocks. The BMI was evaluated by dividing the body weight (in kilograms) by height (in meters) squared (kg/m2). The waist to hip ratio (WHR) was calculated as the ratio of waist circumference over the hip circumference. WHR was calculated as the ratio of waist circumference over the height.

### Semen analysis

Semen samples were obtained after 2–5 days of sexual abstinence and allowed to liquefy for 30 min at 37°. When semen liquefaction was completed, sperm progressive motility, sperm morphology, and semen volume were measured according to the guideline established by WHO in 2010. The CASA system [SPERM CLASS ANALYZER software (SCATM, Microptic, Version 4.2, Barcelona, Spain)] was applied to assess sperm progression, overall motility, and sperm concentration. Spermatozoa were classified as progressive motile (WHO class A + B), non-progressive motile (class C), and immotile (class D). Papanicolaou staining was employed for the analysis of sperm morphology.

### Clinical and biochemical assessment

Biochemical markers, such as serum glucose concentrations were measured using a standard enzymatic method (Roche Diagnostics GmbH, Mannheim, Germany). Serum insulin level was determined using the Electro-chemiluminescence immunoassay (ECLIA) kit (Roche Diagnostics GmbH, Mannheim, Germany). Glycosylated hemoglobin (HbA1C (was analyzed by the Nyco Card Reader II analyzer according to the manufacturer's instructions. Homeostasis model assessment of insulin resistance (HOMA-IR) was assessed according to the following formula: fasting insulin (microU/L) x fasting glucose (nmol/L)/22.5. Serum triglyceride (TG), cholesterol, and high-density lipoprotein (HDL) levels were evaluated by enzymatic methods (Biorex). Serum concentrations of low-density lipoprotein (LDL) and very low-density lipoprotein (VLDL) were determined.

## Reproductive hormones

After overnight fasting, 10 ml of peripheral blood was taken by a trained nurse between 8:00 and 10:00 AM. Serum samples were separated by centrifugation at 3000 g for 15 min, and stored at -80 °C until usage. The concentrations of luteinizing hormone (LH) and follicle-stimulating hormone (FSH) were measured using commercial ELISA kits (Pishtaz Teb Inc., Tehran, Iran). The levels of Total Testosterone (TT) and Serum-free testosterone (FT) were assessed by a commercial ELISA kit (AccuBind ELISA Microwells, Monobina Inc., Lake Forest, CA, USA). Sex hormone-binding globulin (SHBG) levels were analyzed by the electro-chemiluminescence immunoassay (ECLIA) kit.

### Determination of serum pro-inflammatory cytokines

Serum leptin was analyzed by the Human Leptin ELISA Kit (RayBiotech, Inc., Norcross, Georgia, USA). Proinflammatory cytokines, including IL-1, IL-6, and TNF-α were quantified using commercial ELISA kits (RayBiotech, Inc., Norcross, Georgia, USA) according to the manufacturer’s instructions. High-sensitivity C-reactive protein (hs-CRP) levels were evaluated using the CRP kit (pars Azmoon Kit, Tehran, Iran).

### Statistical analyses

Data were presented as the means and standard error of the mean (mean ± SEM). All statistical analyses were performed by the SPSS software (version 16; SPSS, Chicago, ILL). The Kolmogorov–Smirnov test was utilized to examine whether the data were normally distributed. In the case of normal distribution One-way analysis of variance (ANOVA) was applied followed by DunnettT3 post hoc test); otherwise, the Kruskal–Wallis test was employed followed by Bonferroni Correction post hoc test. The correlations among lipids profiles, sperm parameters, and inflammatory factors were calculated by Pearson's correlation coefficient test. The *p*-value.

of less than 0.05 was considered statistically significant.

### Results

A total of 150 men entered to this study. The subjects were classified into four groups based on obesity and diabetes status (40 men with normal weight who were non-diabetic as controls, 35 men with normal weight who were afflicted with diabetes as lean DM group, 40 obese men who were non-diabetic as obese group, and 40 obese men who were affected by diabetes mellitus as obese-DM group). Table 1 presents the results regarding comparison of clinical parameters among these four groups. The four groups were comparable regarding age, BMI, waist and hip circumference. Statistically higher mean age was observed in lean and obese DM groups compared to obese and control groups (*p = *0.001).

**Table 1:** Clinical and biochemical parameters of the study population

Variables	GROUPS				
	Control (*n =* 40)	Lean DM (*n =* 35)	Obese (*n =* 40)	Obese DM (*n =* 35)	*P*-value*
**Clinical** **mean ± SD**					
** Age (years)**	32.78 ± 6.19	39.00 ± 6.28	32.7 ± 6.16	38.31 ± 5.77	0.001
** BMI (kg/m2)**	23.39 ± 1.37	26.41 ± 2.39	36.07 ± 5.43	34.26 ± 4.03	0.001
** WC (cm)**	88.43 ± 4.62	95.86 ± 7.3284	116.4 ± 13.19	113.09 ± 10.06	0.001
** WHR**	0.91 ± 0.043	1.19 ± 0.24	1.25 ± 0.24	0.99 ± 0.06	0.446
** Hip circumference (cm)**	96.27 ± 4.34	97.91 ± 15.83	112.93 ± 20.49	114.23 ± 7.82	0.001

*BMI* Body mass index, *WC* Waist circumference, *WHR* waist-to-hip ratio

**Fig. 1:**
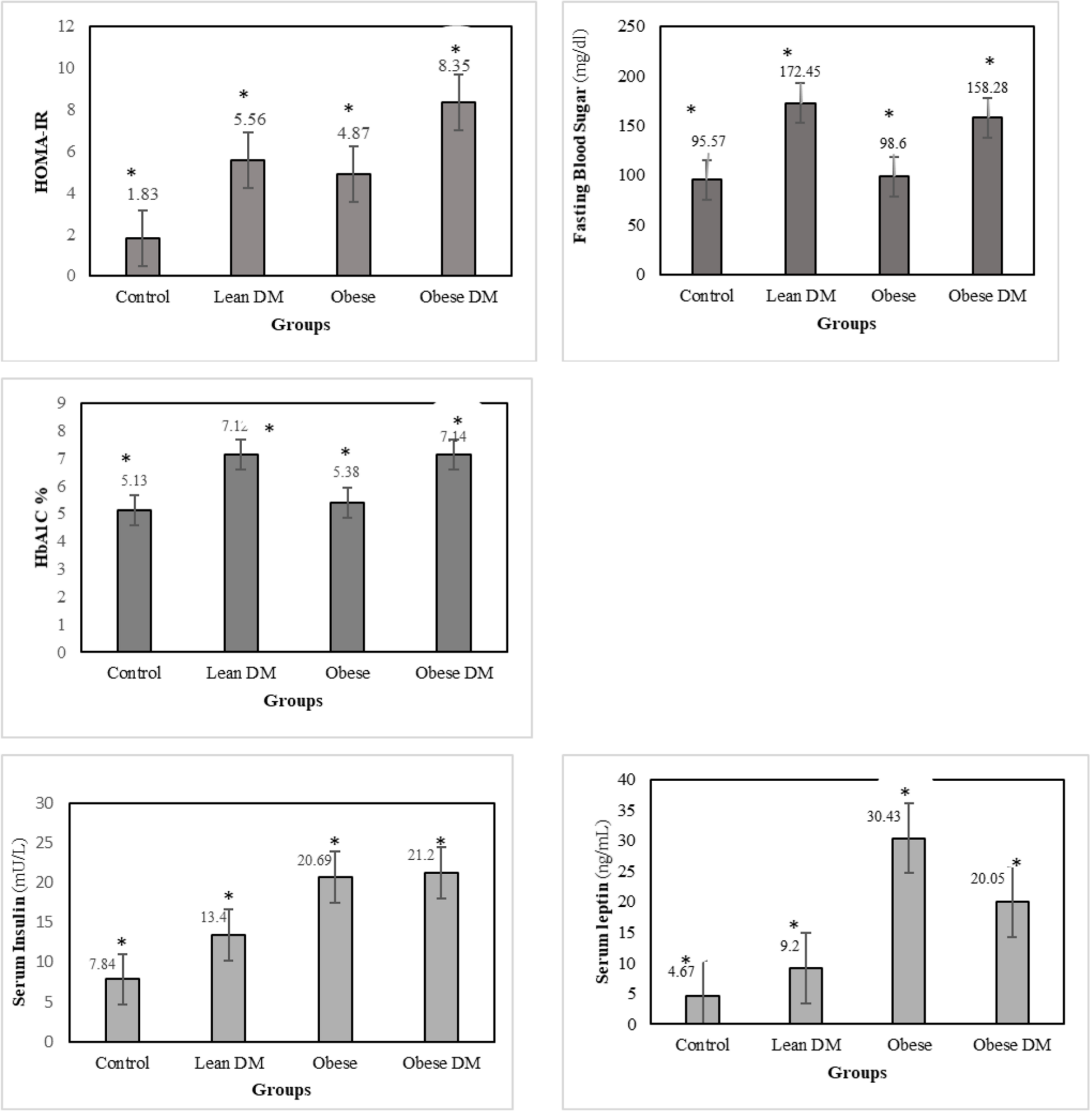
FBS: fasting blood sugar; HbA1C: glycosylated hemoglobin; HOMA-IR homeostasis model assessment of insulin resistance. *Obtained by One-way analysis of variance (ANOVA) test at significant level of 0.05

**Fig. 2:**
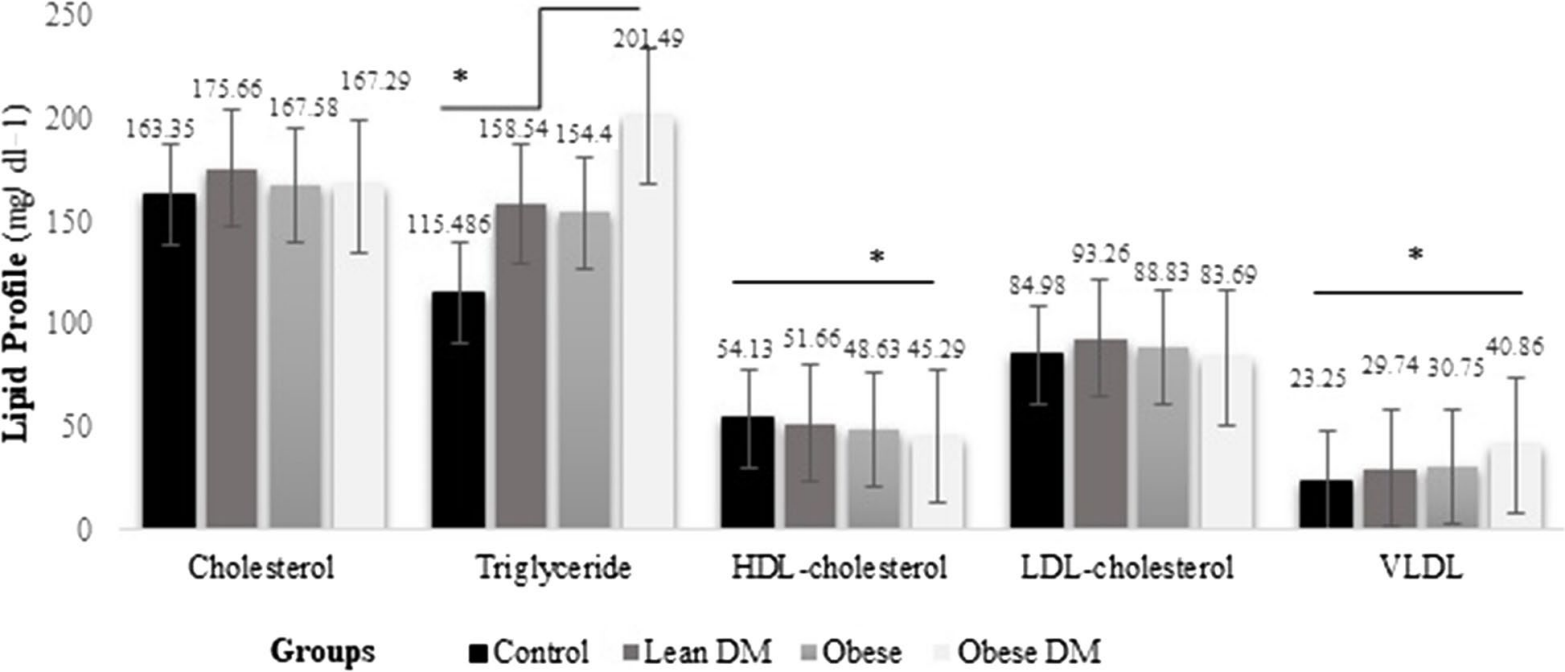
HDL: high-density lipoprotein; LDL: low-density lipoprotein. VLDL: Very low-density lipoprotein. *Obtained by One-way analysis of variance (ANOVA) test at significant level of 0.05

Figure. 1 presents the results regarding comparison of biochemical parameters among these four groups. The four groups were comparable regarding, FBS, HbA1C, HOMA-IR, serum insulin, serum leptin, triglyceride, HDL-cholesterol, and VLDL. There is a significant change in serum insulin between obese and diabetic obese, groups and lean DM and control groups (*p = *0.02 and *p = *0.001, respectively; ANOVA followed by Tukey's post hoc test). There was a significant difference observed in serum leptin between obese and diabetic obese groups, and lean DM and control groups (*p = *0.001, and *p = *0.016, respectively; ANOVA followed by Tukey's pos hoc test). There were higher mean Triglyceride levels in diabetic obese men compared with control group (*p = *0.004). In contrast, there were lower mean HDL-cholesterol levels in diabetic obese men compared with control group (*p = *0.021) (Fig. 2).

### Hormones and semen parameters

Sperm parameters and hormone concentrations were depicted in Table 2. There was a significant difference among these four groups by sperm parameters. Post hoc Tukey simultaneous tests indicated that sperm motility, progressive motility, sperm count, and sperm morphology were significantly lower in obese-DM (*p = *0.001), lean DM (*p = *0.01), and obese groups (*p = *0.001) compared with the control group. No significant difference was found in hormone concentrations except for SHBG. Post hoc Tukey simultaneous tests revealed that the control group had statistically significantly higher mean SHBG than obese-DM (*p = *0.001) and obese groups (*p = *0.011).

**Table 2:** The comparison of sperm parameters and hormone concentrations among four experimental groups

**Variables**	**GROUPS**				
	Control (*n =* 40)	Lean DM (*n =* 35)	Obese (*n =* 40)	Obese DM (*n =* 35)	*P*-value*
**Sperm motility (%)**	74.19 ± 2.42	56.034 ± 4.38	55.71 ± 3.70	54.84 ± 3.83	0.000
**Normal sperm morphology (%)**	28.50 ± 1.75	11.66 ± 1.47	14.03 ± 2.04	16.69 ± 2.09	0.000
**Progressive motility (%)**	49.08 ± 2.79	37.02 ± 3.73	37.56 ± 3.74	33.39 ± 3.28	0.008
**Total normal progressively motile sperm count (n)**	71.41 ± 24.97	47.37 ± 5.16	62.23 ± 3.34a	49.96 ± 5.49	0.003
**FSH (mIU/mL**	3.35 ± 0.36	3.47 ± 0.389	3.41 ± 0.43	3.56 ± 0.34	0.983
**LH (mIU/mL)**	2.49 ± 0.19	2.74 ± 0.20	3.21 ± 0.80	2.53 ± 0.17	0.657
**TT (ng/mL)**	4.47 ± 0.29	3.52 ± 0.23	3.78 ± 0.26	3.70 ± 0.43	0.383
**FT (ng/mL)**	26.41 ± 14.83	15.96 ± 5.34	11.09 ± 0.71	11.38 ± 1.20	0.512
**SHBG**	41.77 ± 2.21	34.06 ± 2.26	32.95 ± 1.96	25.00 ± 2.16	0.000

*FSH* Follicle-stimulating hormone, *LH* Luteinizing hormone, *T* Testosterone, *FT* Free testosterone, *SHBG* Sex hormone-binding globulin

^*^Obtained by One-way analysis of variance (ANOVA) test at significant level of 0.05

### Pro-inflammatory cytokines

**Fig. 3:**
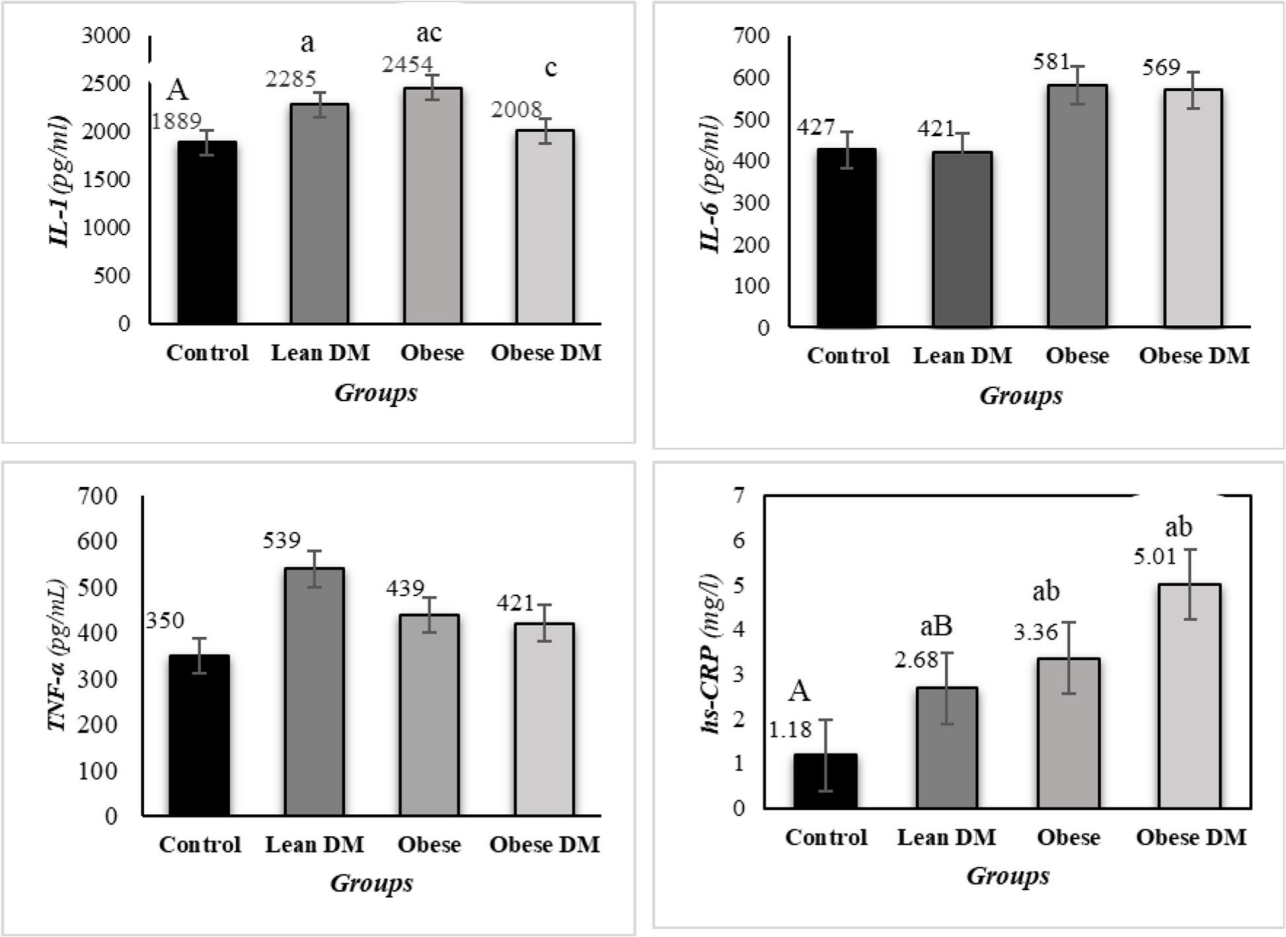
The comparison of hs-CRP, interleukin (IL)-1, IL-6 and TNF-α levels among all experimental groups. Hs-CRP: high-sensitivity C-reactive protein; IL-6, interleukin-6; IL-1: interleukin-1; TNF- α: tumor necrosis factor-alpha. Uppercase letters vs. the same lowercase letters (A vs. a, B vs. b, and C vs. c) indicate a statistically significant difference (p < 0.05)

The comparison of pro-inflammatory cytokines among four groups is shown in Fig. 3. There was a marked difference in the concentration of hs-CRP when all of the four groups were compared, the highest level of hs-CRP were assessed in obese DM. There was a significant difference between obese and diabetic obese groups, and control groups (*p = *0.000, and *p = *0.014, respectively; ANOVA followed by Tukey's pos hoc test). Moreover, serum leptin, which is considered an adipokine was significantly higher in obese-DM, lean DM, and obese groups in comparison with the control group. There was a significant difference between obese and diabetic obese groups, and lean DM and control groups (*p = *0.000, and *p = *0.016, respectively; ANOVA followed by Tukey's pos hoc test) Fig. 1.

## Discussion

Accumulative evidence shows the association of male reproductive dysfunction with the development of obesity and diabetes [8, 20, 21].Hyperinsulinemia and hyperglycemia are common metabolic disorders usually detected in obese and diabetic men, having adverse effects on sperm quantity and quality [22]. Previous investigations reported that obesity and diabetes were associated with reproductive health problem and sub fertility [23, 24]. The results achieved in this study showed that disturbances in sperm parameters in men were probably due to overweight and overall obesity and diabetes.

Our finding indicated the increased levels of serum FBS, HbA1c, insulin, and insulin resistance, as well as older age, in diabetic men. Also, two obese groups had higher BMI and waist circumference values compared with other groups. The values of WHR, serum triglyceride, and VLDL were substantially lower in the control group when compared with other experimental groups. Also, obese DM group had higher levels of triglyceride and VLDL than lean DM and obese groups.

Similar to our results, some studies reported higher values of BMI, waist circumference in diabetic patients in comparison with healthy subjects. Diabetes mellitus has been linked with the lower semen parameters, including total sperm count, normal sperm morphology, sperm motility, and sperm progression [25, 26]. It seems that secondary hypogonadism occurs in obese and diabetic population [10, 27, 28], thus it is not surprising that in present study The present study showed the highest levels of serum testosterone and SHBG in the control group. Previously, higher prevalence of hypogonadism has been reported in patients with type 2 diabetes mellitus, ranging from 4 to 45% [29–32], along with obese individuals, ranging from 15 to 78% [33]. Serum testosterone in obesity and diabetes is commonly associated with the development of secondary hypogonadism [10]. The lower levels of testosterone and SHBG are linked with obesity and higher values of BMI [34]. As a putative mechanism, Aromatization of testosterone occurs in the adipose tissue, deriving the peripheral conversion of testosterone into estradiol [35].

Most of studies have primarily focused on the correlation of increased BMI, adiposity [15], and endocrine dysfunction with the semen quality [6, 36, 37]. Thus, both biochemical and hormonal mechanisms contribute to sperm damages in diabetic and obese men.

We assessed inflammatory biomarkers as another possible mechanism. Our data demonstrated that hs-CRP levels were significantly higher in obese, obese DM and lean DM than controls. Besides, the obese DM group had significantly higher levels of insulin and insulin resistance in comparison with other groups. It seems that adiposity and hyperglycemia alone or in combination are critical factors in the increase of the levels of insulin, insulin resistance, and acute inflammatory biomarkers [38–40].

The current study showed that the serum concentration of IL-1β was significantly increased in lean DM and obese groups compared with the control and obese DM groups; however, there were no significant differences in serum concentrations of IL-6 and TNF-α among four experimental groups. In agreement with other studies, we found that obesity, hyperglycemia, and metabolic disturbance may be associated with the alternation of inflammatory pathways and reproductive health problem [41–43].

The present data demonstrated a positive correlation of metabolic factors, including FBS, HbA1c, insulin resistance, waist circumference, and BMI with serum hs-CRP, leptin levels, and the semen parameters. It appears that obesity could modify inflammatory biomarkers and immune system activity [17, 44–46] directly or indirectly, in addition, the inflammatory factors, such as hs-CRP and leptin may lead to the altered metabolic indices. Adipocytes are responsible for the production of leptin [47, 48]. Higher levels of serum leptin were observed in two obese groups and this hormone per se influences the secretion of LH and FSH from the pituitary, leading to changes in the amplitude of the released pulses, as well as the pulsatility. Therefore Inflammation can harm affects the balance of the HPG axis as a result of presence extra adipose tissue [49]. Finally, metabolic alternations may affect reproductive health and the semen parameters [3, 50] In an experimental study conducted by Fan et al. concluded that the chronic inflammatory status leads to the impairment of the reproductive system in obese [17, 51].

Recently, Condorelli and colleagues investigated several pathophysiological factors on the sperm function in men with type 1 and type 2 diabetes. They suggested the involvement of two main putative mechanisms, including an inflammatory condition accompanied by increased oxidative stress and sperm DNA fragmentation in type 2 diabetes mellitus, along with mitochondrial damages in type 1 diabetes [7, 52].

The present study assessed the association of three mechanisms (hormonal, biochemical and inflammatory) with male sub fertility [7]. Nevertheless, the current research had several limitations, such as lack of the assessment of other pro inflammatory markers. Besides, the present study assessed pro inflammatory cytokines only in blood samples and It is recommended that inflammatory biomarkers be examined in other tissues [53, 54], which shed more light on inflammatory and male infertility.

## Conclusion

The results of this study indicated the association of metabolic changes and hormonal dysfunction. Our findings showed that the obese and diabetic men with higher inflammatory parameters had met worse basic sperm parameters compared to controls. Inflammatory markers may be possible diagnostic value in male infertility adding to other mechanisms. It seems that obesity and diabetes negatively affect the male reproductive organ through the detrimental changes in all three mechanisms mentioned earlier, which warrants further studies.

## Data Availability

Please contact author for data requests.

## Abbreviations

DM: Type 2 diabetes mellitus
HPG: Hypothalamus Pituitary Gonadal
ROS: Reactive oxygen species
WC: Waist circumference
HC: Hip circumference
BMI: Body mass index
WHR: Waist to hip ratio
WHO: World Health Organization
HbA1C: Hemoglobin A1c
HOMR-IR: Homeostatic Model Assessment for Insulin Resistance
TG: Serum triglyceride
HDL: High-density lipoprotein
LDL: Low-density lipoprotein
VLDL: Very low-density lipoprotein
LH: Luteinizing hormone
FSH: Follicle-stimulating hormone
TT: Total Testosterone
FT: Serum-free testosterone
SHBG: Sex hormone-binding globulin
SPSS: Statistical Package for Social ScienceS
